# Ultrastructural and Functional Properties of a Giant Synapse Driving the Piriform Cortex to Mediodorsal Thalamus Projection

**DOI:** 10.3389/fnsyn.2017.00003

**Published:** 2017-01-31

**Authors:** Patric Pelzer, Heinz Horstmann, Thomas Kuner

**Affiliations:** Department of Functional Neuroanatomy, Heidelberg UniversityHeidelberg, Germany

**Keywords:** piriform cortex, mediodorsal thalamus, giant driver synapse, class I synapse, cortico-thalamo-cortical loops, cortico-thalamic synapse, giant synapse, synaptic transmission

## Abstract

Neocortico-thalamo-cortical loops represent a common, yet poorly understood, circuit employing giant synapses also referred to as “class I”, giant, or driver synapses. Here, we characterize a giant synapse formed by projection neurons of the paleocortical piriform cortex (PIR) onto neurons of the mediodorsal thalamus (MD). Three-dimensional (3D) ultrastructure of labeled PIR-MD terminals, obtained by using serial-section scanning electron microscopy (EM) combined with photooxidation-based detection of labeled terminals, revealed a large terminal engulfing multiple postsynaptic dendritic excrescences. The terminal contained multiple synaptic contacts, a high density of synaptic vesicles and several central mitochondria. Using targeted stimulations of single identified PIR-MD terminals in combination with patch-clamp recordings from the connected MD neuron, we found large postsynaptic currents with fast kinetics and strong short-term depression, yet fast recovery upon repetitive stimulation. We conclude that the phylogenetically old paleocortex already developed giant synaptic connections exhibiting similar functional properties as connections formed by giant neocortico-thalamic projections.

## Introduction

Cortico-thalamo-cortical loops operating giant synaptic connections between cortex and thalamus are a characteristic feature of primary sensory cortices and have also been described for associative cortices (Sherman and Guillery, 2006, 2011). While their systemic function remains elusive, these giant synapses have been first reported in electron microscopic studies (Hoogland et al., 1991; Kuroda and Price, 1991). Thalamic giant synapses originating from neocortical areas have been characterized functionally (Turner and Salt, 1998; Li et al., 2003; Reichova and Sherman, 2004; Groh et al., 2008; Seol and Kuner, 2015), while the same synapse type originating from the phylogenetically older paleocortex have not yet been investigated on the level of single identified synaptic connections. Here, we address the question if these, in the phylogenetical context, first giant synaptic connections between piriform cortex (PIR) and mediodorsal thalamus (MD) share similar features with giant synapses formed by neocortical areas. Furthermore, this connection could be relevant for higher cortical processing of olfactory information (Slotnick and Risser, 1990; Kuroda and Price, 1991; Plailly et al., 2008), yet, the role of PIR-MD giant synapses in the olfactory circuit is currently not understood (reviewed in Courtiol and Wilson, 2015).

In general, axonal projections reaching the thalamus may terminate as one of two classes of thalamic synapses: “class I” or “class II” (Sherman and Guillery, 2006). “Class I” synapses convey the principal information and represent the neurons receptive field (Sherman and Guillery, 2011). “Class II” synapses influence the neurons input-output function to “class I” inputs (Sherman and Guillery, 2011). They do so by shifting the resting membrane potential and thus changing the availability of T-Type low-threshold activated calcium-channels (Mease et al., 2014). This I_T_ current can be triggered by synaptic inputs, if the neuron is relatively hyperpolarized. On top of the depolarization lasting up to hundreds of milliseconds, also referred to as the low threshold calcium spike, ride multiple high frequency action potentials (APs)—the burst, while at more depolarized potentials, the neuron usually responds to individual synaptic inputs with only a single AP—the tonic mode (Llinás and Jahnsen, 1982).

Neurons in the deep layers of the PIR (reviewed in Courtiol and Wilson, 2015) have been found to form glutamatergic synapses consisting of both RL-type (round vesicles, large) and RS-type (round vesicles, small) boutons as determined with electron microscopy (EM; Kuroda and Price, 1991; Kuroda et al., 1992). RL-type boutons are the ultrastructural correlate of “class I” synapses, while RS-types correspond to “class II” synapses. The functional properties of PIR-MD RL-type boutons have not yet been revealed, yet, are crucial to know with regard to their potential physiological roles. While RL-type profiles in MD have been described by Kuroda and Price (1991) in single representative ultrathin sections, a three-dimensional (3D) morphology description of corticothalamic giant synapses has only been shown for a RL-type synapse connecting cortical layer 5b neurons of the somatosensory cortex with relay neurons of the posteromedial nucleus (POm; Hoogland et al., 1991). These boutons are characterized by a large diameter (>2 μm), multiple synaptic contacts, multiple mitochondria, round synaptic vesicles, and sometimes multivesicular bodies. On the dendritic side invaginations, i.e., excrescences, are a characteristic hallmark. These Lb5-POm RL-type boutons have been characterized by targeted stimulation of identified terminals (Groh et al., 2008; Seol and Kuner, 2015). They exhibit large excitatory postsynaptic currents (EPSC) and utilize mainly AMPA receptor GluA4 subunits to mediate the fast component of the EPSC (Seol and Kuner, 2015). The synapse exhibits a strong use-dependent depression that may act as a functional switch between a driver and coincidence detector mode of function (Groh et al., 2008). A fully recovered synapse may evoke postsynaptic APs, while during a stimulus train, mimicking *in vivo* activity, only two precisely co-occurring inputs sum to suprathreshold levels. Thus, knowing the properties of and potential differences between different cortico-thalamic synapses will be crucial to understand their specific contributions to the function of cortico-thalamo-cortical loops.

We here validated the hypothesis that a proportion of PIR-MD synapses are functionally “class I” synapses by recording single synaptically evoked EPSCs in an acute brain slice preparation. To this end, we utilized gene transfer via adenoassociated viruses (AAV) to heterologously express a synaptophysin-eGFP fusion protein in neurons of the PIR. The tagged vesicular synaptophysin enabled us to identify PIR-synapses within the MD. Via a juxtaposed double-barrel stimulation-electrode we evoked presynaptic transmitter release in a repetitive stimulation paradigm from identified PIR-MD giant synapses. Furthermore, we used the photooxidation method (Horstmann et al., 2013) to identify and reconstruct electron microscopic 3D models of PIR synapses. According to the classification criteria proposed by Sherman and Guillery (2006), a proportion of PIR-MD synapses fulfills the requirements in all measured parameters to be classified as “class I” synapses. We found large EPSC amplitudes, fast short-term depression and rapid recovery from depression in recordings from individually stimulated PIR-MD synapses.

## Materials and Methods

### Animals

Wild-type (C57Bl/6N) mice were housed in individually ventilated cages under defined housing conditions (12 h-12 h day-night cycle, 55 ± 5% humidity and *ad libitum* access to food and water) with their mother until the final experiment was done. The animals reached a maximum age of P48. All experiments were conducted in accordance with the German animal welfare guidelines and were approved by the responsible authority (Regierungspräsidium Karlsruhe).

### Plasmids and Adenoassociated Virus (AAV) Production

All used constructs have been described previously (Wimmer et al., 2004; Horstmann et al., 2013). The cDNA of the synaptically enriched vesicular synaptophysin protein was fused with eGFP or two copies of pHluorin (Miesenböck et al., 1998), respectively. The fusion proteins were subcloned into the pAM vector backbone, consisting of the 1.1 kb long joint cytomegalovirus enhancer sequence and chicken β-actin promoter, the woodchuck post-transcriptional regulatory element and the bovine growth hormone polyA signal. Recombinant AAV chimeric particles (helper plasmids for AAV1 and AAV2 capsids at 1:1 ratio) were extracted from human embryonic kidney (HEK) 293 cells 3 days after transfection via the calcium phosphate precipitation (During et al., 2003; Grimm et al., 2003). The virus particles were purified from the lysate via a heparin-agarose Typ I column (Cat. No: 7321010, Biorad, Hercules, CA, USA).

### Stereotaxic Injection

Stereotaxic procedures have been described previously (Wimmer et al., 2004; Groh et al., 2008; Seol and Kuner, 2015). In brief: young mice (P 14) received an initial anesthesia with 5% isoflurane in O_2_ carrier gas, while it was maintained with 1.5% isoflurane. To ensure analgesia after the surgery, mice were subcutaneously injected with Carprofen (10 mg/kg body weight, product name: Rimadyl, Pfizer, Berlin, Germany), preheated to 37°C. The animals head was fixed in a stereotaxic alignment system (David Kopf Instruments, Tujunga, CA, USA). Upon leveling, craniectomies were performed with a dental drill (EXL-40, Osada, Los Angeles, CA, USA). The virus particles were injected into the right PIR at the following x, y, z coordinates in mm from Bregma: (1) 3.3, 2.3, −4.7; (2) 3.7, 2.1, 4.7; (3) 4.1, 1.9, 4.7–5.0; (4) 4.5, 1.7, 5.0; (5) 4.9, 1.5, 4.8–5.1; (6) 5.0, 1.1, 4.8–5.1; (7) 5.1, 0.7, 4.8–5.1 (where the z-axis is angled by 27° parallel to the mediolateral axis; the z-coordinates ranges are the beginning and end of a trajectory). A total of 138 nL was injected using the Nanoject II (Drummond Scientific Company, Broomall, PA, USA). The injection volume typically covered the deep layer III and endopiriform cortex. The layer II neurons are partially included at a variable degree. The injection volume may at times also extend across the borders to the olfactory tubercle and agranular insular cortex. Along the anterior-posterior axis the injections ranged from the anterior pole of the PIR to a site situated 0.7 mm anterior of Bregma. During the initial two post-surgery days, analgesia was maintained with additional, daily Carprofen injections.

### Photooxidation

Photooxidation-mediated specific labeling of GFP-expressing neurons for EM was done as described by Horstmann et al. (2013). Briefly, perfusion-fixed (4% PFA) brain slices from animals injected with AAV-synaptophysin-2-pHluorin were cut at 200 μm thickness. The slice of interest was incubated in an oxygenated Tris-HCl buffer overnight. The following day, the pHluorin was excited under a light microscope in a 1 mg/mL 3,3′-Diaminobenzidine (DAB) supplemented Tris-HCl buffer. The subsequently excised region of interest with the DAB-precipitate was embedded in epoxy resin. For EM the tissue block was serially sectioned at a thicknes of 38 nm using an Ultracut S ultramicrotome (Leica, Germany) equipped with a diamond knife angled at 35° (Diatome, Biel, Germany). The 109 sections were collected onto hydrophilized silicon wafer strips and post stained with Reynolds lead citrate.

### Electron Microscopy

The sections were imaged on a Zeiss 1530 (Zeiss, Germany) scanning electron microscope. The InLens detector was used at a working distance of 2.1 mm and 3 keV acceleration voltage (Horstmann et al., 2013). A total area of 11.6 × 8.7 μm at a 10,000× magnification was imaged at a resolution of 3072 × 2304 pixel.

### Preparation of Acute Slices

Acute slices from mice injected with AAV-synaptophysin-GFP were prepared 12–37 days post-surgery, as described before (Groh et al., 2008; Seol and Kuner, 2015). Slices of 250–300 μm thickness were cut with a vibratome (VT1200 S, Leica, Germany) in ice-cold solution containing (in mM): 85 NaCl, 2.5 KCl, 10 glucose, 75 sucrose, 25 NaHCO_3_, 1.25 NaH_2_PO_4_, 3 MgCl_2_, 0.1 CaCl_2_, 3 3-myo-inositol, 2 Na-pyruvate, 0.4 ascorbic acid, aerated with carbogen (5% CO_2_), pH 7.3. After cutting, the slices were transferred for 30 min to 37°C (in mM): 109 NaCl, 4 KCl, 35 glucose, 25 NaHCO_3_, 1.25 NaH_2_PO_4_, 1.3 MgCl_2_, 1.5 CaCl_2_, 3 3-myo-inositol, 2 Na-pyruvate, 0.4 ascoribic acid, aerated with carbogen, pH 7.3. Subsequently slices were kept at room temperature.

### Electrophysiology

Whole-cell patch-clamp recordings were established from MD neurons. All neurons were recorded at room temperature (22 ± 1°C) in artificial cerebrospinal fluid (ACSF, in mM: 125 NaCl, 2.5 KCl, 15 glucose, 25 NaHCO_3_, 1.25 NaH_2_PO_4_, 2 CaCl_2_, 1 MgCl_2_, aerated with carbogen, pH 7.3). The patch pipettes were pulled from borosilicate glass capillaries (1B150F-4, World Precision Instruments, Sarasota, FL, USA) on a horizontal puller (P97, Sutter Instruments, Novato, CA, USA) and had an open tip resistance of 3–7 MΩ. They were backfilled with intracellular solution containing (in mM): 130 K-gluconate, 20 KCl, 5 Na_2_phosphocratine, 10 HEPES, 5 EGTA, 4 Mg-ATP supplemented with Alexa 594 (37 μM and additional 0.73 mM KCl; life technologies, Carlsbad, CA, USA).

All electrophysiological recordings were done with a HEKA EPC-10 amplifier (HEKA Electronics, Lambrecht, Germany), controlled by PatchMaster. The recordings were double low-pass filtered with internal Bessel filters (2.9 kHz and 10 kHz) and digitized at sampling rates of 20 kHz. All neurons were measured in whole-cell voltage-clamp mode at −70 mV unless noted otherwise. The liquid junction potential (13.4 mV, calculated based on Barry and Lynch, 1991) was corrected for. The stimulation electrode was obtained from a double barrel borosilicate glass capillary with a tip opening of 1–2 μm (Cat. No: 1401021, Hilgenberg, Malsfeld, Germany) using the horizontal puller to yield a small and confined electric field around the tip. The current was controlled with an Iso-Stim 01DPI (NPI Electronic Insturments, Tamm, Germany) stimulator with 10–90 V and 10–90 μs pulse width.

We have used the following criteria to select valid recordings of monosynaptic connections between the PIR and the MD: (a) The onset of the postsynaptic current needed to be clearly separated from the stimulus artifact; (b) The postsynaptic response must not scale with the stimulus intensity, but be all-or-none; (c) The current needed to be monosynaptic (This was determined as no further increase in EPSC amplitude upon an increase of stimulation intensity or by inspection for a second (slightly) delayed component. Both events are assumed to result from stimulation of two distinct yet closely spaced synapses within the electric field of the stimulation electrode); (d) The stimulation must be reliable (every stimulus should trigger a postsynaptic response, at least when stimulating at low frequency); (e) The holding potential needed to be stable throughout the recording. These criteria limited the number of synapses that were analyzed: 23 out of 31 synapses from 130 animals were excluded, some more synapses were disqualified already during the ongoing recording. In the majority of attempts successful recordings could not be obtained. Examples for excluded recordings are given in Supplementary Figure S1.

### Image Analysis

#### Dendritic Length Between Stimulated Synapse and Soma

All images were processed with Fiji (Schindelin et al., 2012) and the Simple Neurite Tracer plug-in (Longair et al., 2011). The Simple Neurite Tracer was used to measure the dendritic length between the soma and the synapse. The plug-in searches for the brightest trace (and its length) of voxels in 3D between manually selected spots.

#### Synaptic Volume of Stimulated Synapses

All images were processed with Fiji and additional plug-ins: FindFoci (Herbert et al., 2014) and 3D ROI Manager (Ollion et al., 2013). The FindFoci plug-in was used to find the volume of the synapse. The search parameters were set to auto threshold (mean + 3*x* SD). Above the threshold local maxima were found. Starting from the local maxima all voxels above threshold were included into the volume. To ensure that minor fluctuations in the signal intensity are not scored as individual volumes, the image was first filtered with Gaussian blur with 1 px range. Finally, volumes were merged if the saddle point between two local maxima was higher than 50% of the peak height (peak intensity—threshold) of either peak. Volumes were excluded, if it contained less than five voxels. The segmented image was loaded into the 3D ROI Manger, with which the volume of the synapse of interest was calculated.

### Data Analysis and Statistics

The analysis was done using Igor Pro (v6.2, WaveMetrics Inc., Lake Oswego, OR, USA) with custom written routines. Values are reported as mean ± standard deviation (SD), unless noted otherwise.

## Results

### Labeling PIR-MD Terminals for Ultrastructural and Electrophysiological Analysis

**Figure 1:**
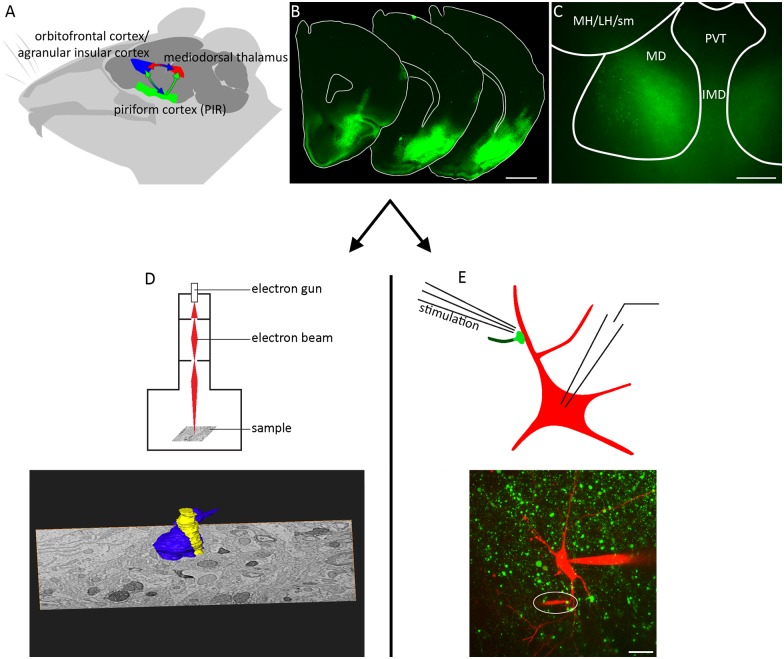
**Injection in the piriform cortex (PIR) and experimental readouts. (A)** The direct and indirect connectivity from PIR to orbitofrontal cortex via the mediodorsal thalamus (MD). Particular emphasis is laid on the connection PIR-MD as this was targeted by **(B)** the local viral-mediated expression of synaptophysin-GFP and **(C)** slicing of the MD. **(D)** Following tissue preparation detailed images of the serial sectioned electron microscopy (EM)-compatible sample were acquired and used for three-dimensional (3D) reconstruction. **(E)** Schematic representation of the electrophysiological recording and stimulation arrangement. The image shows a maximum projection of a confocal image stack with the patch pipette from the right and the tip of the stimulation pipette highlighted by a white ellipse. MH/LH/sm: medial habenula/lateral habenula/stria medullaris, PVT, paraventricular thalamus; IMD, intermediodorsal thalamus; MD, mediodorsal thalamus. Scale bars = **(B)** 500 μm, **(C)** 100 μm, **(E)** 10 μm.

We used spatiotemporally precise gene transfer via viral shuttles to yield heterologous expression of the reporter proteins Synaptophysin-EGFP and Synaptophysin-2pHluorin for electrophysiogical and ultrastructural analyses, respectively (Figure 1). The virus was delivered to the PIR (Figure 1A, green) via stereotactically guided injection needles. Subsequent to the expression period of 12–37 days, expression of the reporter protein was found in the somata of PIR neurons (Figure 1B) and their terminations in the MD (Figure 1C). For EM, mice were transcardially perfusion fixed, the MD region was excised and photooxidation was carried out to reveal PIR-MD synapses for subsequent 3D reconstruction (Figure 1D). For electrophysiology experiments, acute brain slices containing labeled presynaptic PIR-MD were obtained (Figure 1E). The scheme illustrates the recording situation that allows visually targeted stimulation of labeled PIR-MD terminals (green dots in Figure 1E) while performing postsynaptic whole-cell recordings.

### PIR-MD Synapse Ultrastructure

**Figure 2:**
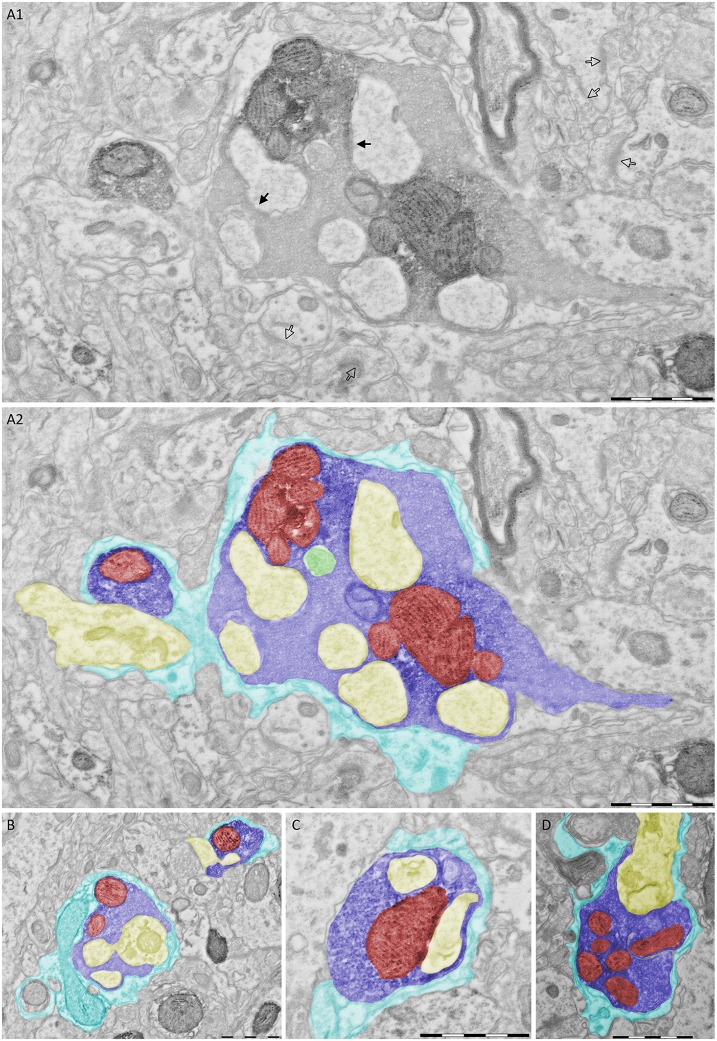
**Piriform cortex-mediodorsal thalamus (PIR-MD) synapse cross-sections. (A1)** Raw image of an electron microscopic cross-section through a labeled RL-type synapse in the MD. In addition, a cross-section of a smaller labeled terminal is visible that could not be classified as RL or RS because only a small portion of it was contained in the tissue block. **(A2)** The same cross-section with important structures color coded (Blue = presynaptic terminal, yellow = post-synaptic dendrite, red = mitochondria, green = multivesicular body, cyan = glial sheet). **(B)** Two more examples of RL-type profiles. Both boutons have multiple mitochondria, although only one is apparent in the smaller bouton in this particular cross-section. In both cases the dendrite has excrescences invading into the bouton, which are fully engulfed. In both cases the bouton is filled with vesicles. Both boutons are ensheathed by the same glial structure. **(C,D)** Two further examples of cross-sections of individual RL-type synapses. Scale bars represent 1 μm.

To compare the synapse to previously published work (Kuroda and Price, 1991) and other thalamic giant synapses we first determined its 3D ultrastructure. PIR-MD terminals were labeled by viral expression of pHluorin-tagged synaptophysin in PIR neurons, allowing for photooxidation-based identification of ultrastructural compartments formed by PIR neurons (Horstmann et al., 2013). The injection site covered the full PIR and in some cases included a minor spill over to the agranular insular cortex and somatosensory cortex. After reaching steady-state expression levels at 21 days post injection, tissue blocks containing the MD were prepared for EM. Within the inspected tissue block we found three RL-type terminals, i.e., morphologically “class I” synapses, that could be fully reconstructed and two further giant terminals that were partially reconstructed. Figure 2A1 shows a representative section containing PIR-MD terminals that can be identified by the increase in gray-scale intensity compared to the surrounding tissue. The section shown in Figure 2A contains abundant amounts of synaptic vesicles, cross-sections of mitochondria (red) and a multivesicular body (green in Figure 2A2). Furthermore, dendritic spine-like protrusions (yellow), also referred to as excrescences, are entirely surrounded by the presynaptic terminal (Figure 2A). Synaptic contacts were difficult to detect reliably, owed to the photooxidation product required to ensure reliable identification of PIR terminals. This is illustrated in Figure 2A, with non-labled synaptic contacts forming readily discernible synaptic contacts (open arrows) while synaptic contacts in the labeled terminal could not be reliably identified (filled arrows show candidate synaptic contacts). This problem is further illustrated and discussed in Supplementary Figures S2, S3. Therefore, we unfortunately could not count and quantify active zones in labeled PIR-MD terminals. For the same reason the quantification of synaptic vesicles would be an underestimate of the true number. It is however evident, that the presynaptic space contains a high density of synaptic vesicles (Figure 2). In all of the five synapses investigated, the terminal is at least partially ensheathed by glial processes, consistent with a glomerular structure. Similar ultrastructural properties were found in all terminals analyzed (Figures 2B–D; *n* = 5 synapses taken from one animal). These features are consistent with previous work describing the ultrastructure of PIR-MD neurons.

**Figure 3:**
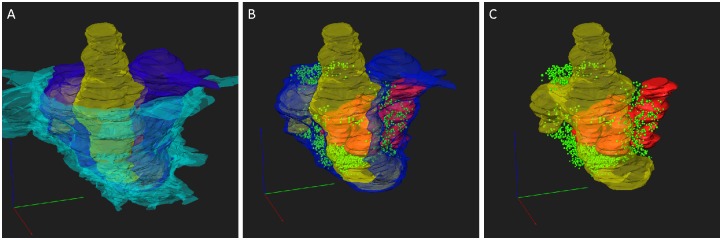
**3D reconstruction of a PIR-MD synapses from serial sections. (A)** The full glomerulus including the glial sheath is depicted. **(B)** The same synapse including the visualization of the mitochondria and synaptic vesicles, lacking the glial sheath. **(C)** Similar visualization without the presynaptic compartment. Cyan = glia sheath, blue = presynaptic terminal, yellow = post-synaptic dendrite, green = vesicles, red = mitochondria. Scale bars = 1 μm.

To address the 3D structure of PIR-MD synapses, we created complete reconstructions of three terminals and partial reconstructions of two terminals. Figure 3 shows the reconstruction of one of these terminals (blue). The entire terminal is covered by astroglia (cyan). Vesicles are distributed in clusters (green). The terminal contains several mitochondria (red) and wraps around the postsynaptic dendrite (yellow). Protrusions (also referred to as excrescences) emanate from the dendrite to increase the contact area between the pre- and post-synaptic compartments. The average volume of the terminals was 1.45 ± 0.76 (2.27 μm^3^, 1.34 μm^3^ and 0.75 μm^3^) with a maximal diameter of 3.5 μm. These synapses contain 6 ± 1.73 (8, 5 and 5) mitochondria respectively that take up 21.47 ± 2.31 (19.93%, 20.35% and 24.12%) of the total volume. The presynaptic profile engulfs the postsynaptic dendrite partially and its spine-like protrusions of the dendrite fully (Figures 2, 3). We also observed labeled synapses that we classified as RS-boutons, the morphological equivalent of “class II” synapses, but did not further analyze them. In conclusion, the large PIR-MD terminals characterized here contain several mitochondria, dendritic excrescences, abundant amounts of synaptic vesicles and several active zones, consistent with a potential function as a driver (class 1) synapse.

### Properties of MD Neurons

We first examined MD neurons contacted by labeled PIR-MD synapses as identified by confocal microscopy. MD neurons had a resting membrane potential (V_rest_) of −75.8 ± 7.1 mV (*n* = 64). The membrane potential (V_m_) change during a 500 ms/−100 pA current injection yields a membrane resistance (R_mem_) of 192.1 ± 68.9 MΩ, according to Ohm’s law. Current depolarizations done from different resting membrane potentials elicited the typical tonic and burst firing modes known for thalamic relay cells (Supplementary Figure S4).

### Evoked Postsynaptic Currents

**Figure 4:**
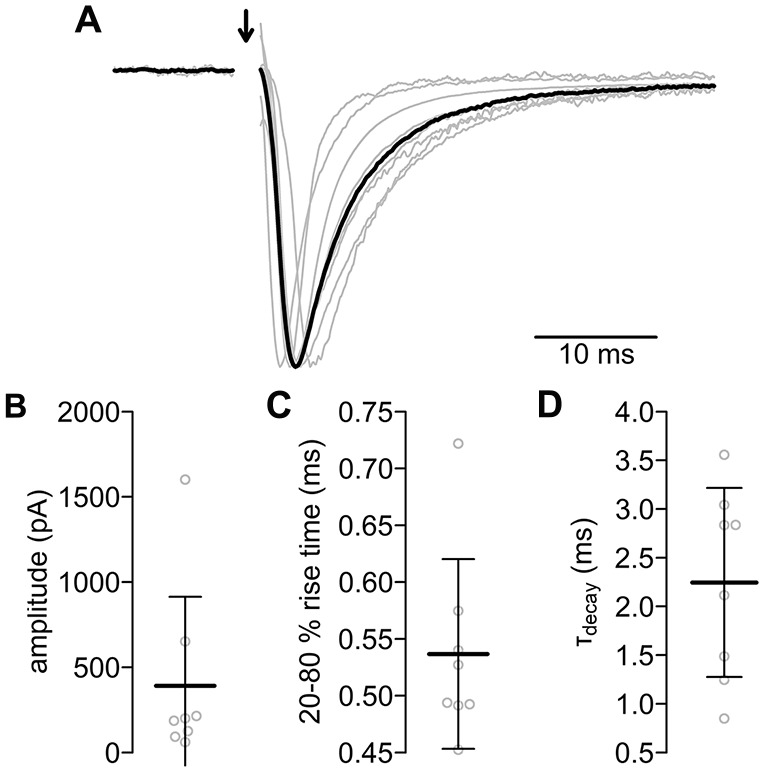
**Evoked postsynaptic currents of the PIR-MD synapses. (A)** All normalized excitatory postsynaptic currents (EPSCs; gray lines) and their average (black). The stimulus artifact was removed (arrow). Scatterplots of **(B)** amplitude, **(C)** rise time and **(D)** decay time constant. Lines are mean ± standard deviation (SD).

We used the approach depicted in Figure 1 to record EPSCs from MD neurons elicited by stimulating defined presynaptic PIR-MD terminals (Figure 4, see “Materials and Methods” Section for criteria defining a successful recording and Supplementary Figure S1). The basic characteristics of the EPSCs evoked by the PIR-MD synapse are shown in Figure 4. The EPSC amplitude was on average 391.2 ± 522.3 pA (*n* = 8). The current rises from 20% to 80% in 0.54 ± 0.08 ms. After the peak, the current decays back to baseline with a time constant of 2.25 ± 0.97 ms. The stimulated synapses were on average 47.0 ± 15.8 μm away from the soma (data not shown). The EPSC amplitude does not correlate significantly with the size of the synapse (Supplementary Figure S5). Moreover, the distance (in the range of up to 80 μm) between synapse and soma is not correlated to the EPSC amplitude or the decay kinetics of the EPSC of this particular synapse (Supplementary Figure S5). Neither did we find correlations of these parameters with somatic access resistance (resistance and amplitude: *r* = 0.00, *p* = 0.99; resistance and decay time: *r* = −0.66, *p* = 0.07; resistance and rise time: *r* = −0.53, *p* = 0.18.). The age of the mice within the span of 29–48 days does neither affect the amplitude, nor the decay kinetics (Supplementary Figure S5). In summary, the EPSC responses evoked by single presynaptic stimulations are consistent with the class I ultrastructure described above.

### Short-Term Plasticity

**Figure 5:**
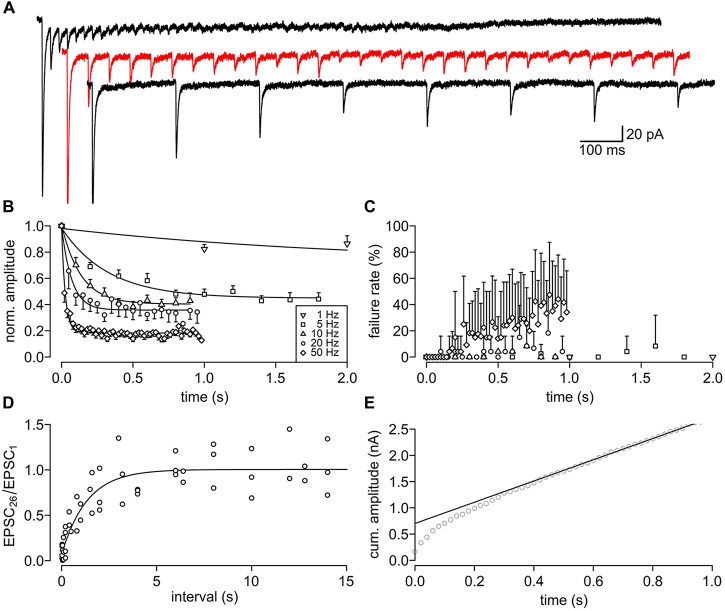
**Short-term depression (STD) and its recovery of the PIR-MD synapse. (A)** Example traces of repetitively (5, 10 and 50 Hz) stimulated EPSCs. **(B)** The normalized amplitudes of EPSCs during trains of stimuli at 1–50 Hz (mean ± SEM (unidirectional), *n* = 8). The depression is fitted monoexponentially. **(C)** Failure rate of stimulation. Symbols as in **(A)** (mean ± SD (unidirectional), *n* = 8). **(D)** The synapses (*n* = 5) were first stimulated 25 times at 50 Hz to deplete the vesicle pool. Following a variable interval an identical test train was applied. The recovery rate is calculated as the amplitude of the first EPSC in the test train (EPSC_26_) normalized by the first EPSC in the depression train (EPSC_1_). Outliers, defined by the 1.5× the interquartile range method, were dismissed. The pooled data is fitted by a monoexponential function (τ = 1.34 ± 0.29 s). **(E)** Back extrapolation per synapse of the readily releasable pool (RRP) from the linear phase of the cumulative amplitude plot (data shown for one synapse).

To examine the synaptic transfer properties upon high frequency stimulation, we stimulated the synapses with trains of stimuli at different frequencies (1, 5, 10, 20, 50, 100 Hz, *n* = 8). We found pronounced short-term depression during repetitive stimulation (Figure 5A). The kinetics and steady state of the depression was frequency-dependent (1 Hz: 2.13 s and 70.9%, 5 Hz: 0.33 s and 44.9%, 10 Hz: 0.14 s and 40.4%, 20 Hz: 0.07 s and 35.8%, 50 Hz: 0.03 s and 18.3%; Figure 5B). The synapse could follow 20 Hz quite reliably, while at 50 Hz the failure rate increases over time, i.e., with every consecutive stimulus (Figure 5C). The failure rate rose up to a maximum of 47.5% within the 1 s stimulus train. The synapses could not follow 100 Hz stimulation (data not shown). The term “short-term plasticity” intrinsically implies that the depression persists only for a short duration. We tested the recovery from depression via two consecutive trains of 25 stimuli at 50 Hz with variable interstimulus intervals of up to 15 s. The recovery of the synapse could be described with a single exponential function (tau = 1.34 ± 0.29 s; Figure 5D). Hence, even short high-frequency trains will cause strong synaptic depression that recovers within a few seconds. These features predict a strong frequency filtering at the PIR-MD synapse.

Furthermore, we estimated the readily releasable pool (RRP) size using the back-extrapolation method (Schneggenburger et al., 1999; Figure 5E). We found an RRP of the PIR-MD synapse of 862.2 ± 947.5 pA (*n* = 8) and a release probability, i.e., the fraction of the RRP released by a single presynaptic AP, of 49.7 ± 21.6%. These values may under- and over-estimate the RRP and release probability, respectively, because postsynaptic factors such as desensitization of ionotropic glutamate receptors are not considered in the analysis. Given the minor influence of postsynaptic factors to short term depression found in another representative of thalamic class I synapses (Groh et al., 2008), we assume that this may also apply to the PID-MD synapse.

### Evoked Postsynaptic Potentials

**Figure 6:**
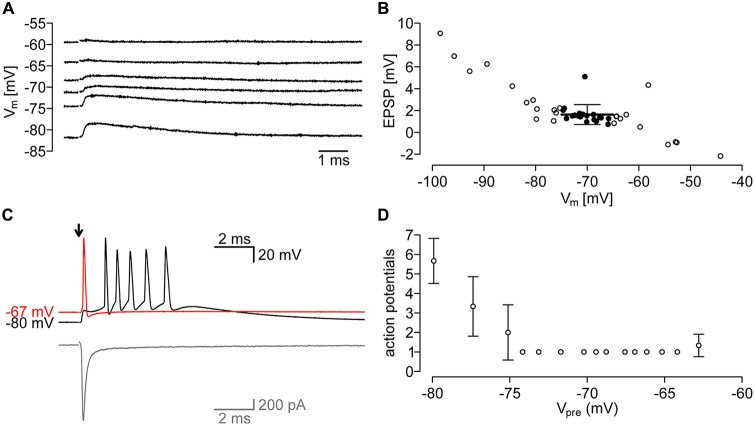
**Evoked postsynaptic potentials of the PIR-MD Synapses. (A)** Example traces of EPSPs at different membrane potentials. **(B)** Quantification of EPSPs at various membrane potentials. Emphasis is laid on EPSP in the range of −75 mV, to −65 mV (filled circles). The average EPSP in that range is 1.63 ± 0.91 mV. **(C)** Example trace of action potentials (APs) evoked through synaptic stimulation at membrane potentials of −80 mV and −67 mV. In gray the corresponding current trace. **(D)** At lower membrane potentials (−75 mV) the number of APs in the AP-burst is dependent on the membrane potential (*n* = 3 stimuli at each potential).

To assess the effect of PIR-MD synapses on the membrane potential of the MD neuron, we recorded in current clamp mode while stimulating presynaptically (Figure 6). We typically found an EPSP of 1.63 ± 0.91 mV (*n* = 6 synapses) at membrane potentials between −75 mV and −65 mV (Figure 6B). We noticed that the sign of the EPSP switched between −60 mV and −55 mV, an unexpectedly negative reversal potential for ionotropic glutamate receptors, opening the possibility of a Cl^−^ conductance contributing to the response. However, this observation is based on four data points recorded from two cells and may not be representative. Nevertheless, owing to difficulties of obtaining a sufficient number of recordings to clarify this, we tested for the occurrence of the vesicular GABA transporter (VGAT) in PIR-MD terminals using targeted immunohistochemistry (Supplementary Figure S6). This analysis shows a strong coexpression of the vesicular glutamate transporter VGLUT-1 with GFP-labeled PIR-MD terminals (Supplementary Figure S6C, blue line), but no coexpression with VGAT (red line), supporting our conclusion that PIR-MD terminals are glutamatergic.

We further attempted to record postsynaptic suprathreshold responses elicited by single presynaptic APs, however, we succeeded only in a single recording (Figure 6C). The number of APs generated depended on the resting membrane potential, with several APs elicited at a membrane potential of −80 mV and only a single AP at more depolarized membrane potentials (Figure 6D). Unfortunately, we could not achieve additional recordings exhibiting suprathreshold responses, so that the results shown here need to be considered preliminary. Nevertheless, the response properties of this cell are very similar to that found in neocortical L5b-POm terminals (Groh et al., 2008; Seol and Kuner, 2015).

## Discussion

### PIR-MD Synapse Ultrastructure

Our work revealed the 3D ultrastructure of labeled PIR-MD terminals. The terminals have a large volume, are vesicle filled, harbor multiple mitochondria, may contain multivesicular bodies, engulf excrescences of the dendrite and a glial wrap. This is consistent with early work of Spacek and Lieberman (1974), who provided the first 3D-ultrastructure of synaptic glomeruli in the rat ventrobasal complex of the thalamus, although not knowing the identity of the terminals. They also found the complex architecture of excrescences engulfed by the presynaptic bouton. Within their reconstructed boutons, the mitochondria made up a smaller proportion of the lumen, i.e., only 11.6% instead of the 21.5 ± 2.3% found here. However, it is not clear if their percentage is proportional to the presynaptic volume, as it is here, or proportional to the combined presynaptic and postsynaptic excrescences volume. Besides representing a true difference, it could also arise from different fixation and processing methods.

Budisantoso et al. (2012) found a similar 3D organization at the retinogeniculate synapse. Multiple protrusions grow out of the dendrite to enlarge the contact surface between bouton and dendrite. They quantified 27 ± 2.7 synaptic contacts per bouton. They concluded that this comes at the expense or as a feature of short-term depression, as the surplus glutamate cannot rapidly diffuse and is not cleared away, but will enhance AMPA receptor desensitization. On the contrary, the stronger argument against desensitization during STD was raised by the use of kynurenic acid, a low affinity antagonist for AMPA receptors, which reduces desensitization and saturation (Scheuss et al., 2002; Wong et al., 2003), at the L5B-POm synapse (Groh et al., 2008).

In summary, the giant PIR-MD synapse resembles the same ultrastructural features as previously described synapses of the class.

### Are PIR-MD Synapses “Class I”?

According to the list of criteria proposed by Sherman and Guillery (2006) class I synapses need to be large. The 3D reconstructions of the synapse from EM images clearly show the hallmarks of RL-type synapses: large diameter, round vesicles, multiple mitochondria, and excrescences. Furthermore, “class I” synapses are supposed to have large EPSCs (reflecting the release of multiple quanta from many active zones), exhibit marked paired-pulse depression, are glutamatergic and activate ionotropic receptors. Similar EPSC kinetics were found in another “class I” synapse with pharmacologic and molecular properties typical for a glutamatergic synapse (Table 1). This indicates that the PIR-MD synapse is glutamatergic and acts on fast ionotropic receptors. Additional indication that glutamate is the principal neurotransmitter stems from tracing studies (Kuroda and Price, 1991; Ray and Price, 1992) and the ultrastructural observation that round synaptic vesicles typically contain the neurotransmitter glutamate. The amplitude of the EPSC is on average larger than for the L5B-POm synapse in mice (Table 1; Seol and Kuner, 2015). “Class I” synapses also show above mentioned paired-pulse depression, a feature strongly expressed in PIR-MD synapses (Figure 5). The EPSC amplitude depresses in a stimulation frequency-dependent manner, as it has been described for other “class I” synapses (Turner and Salt, 1998; Li et al., 2003; Reichova and Sherman, 2004; Groh et al., 2008; Budisantoso et al., 2012; Seol and Kuner, 2015). Additionally, “class I” synapses exhibit a high release probability. The PIR-MD synapses match release probabilities (P_rel_) of L5B-POm synapses for mice, but have a lower P_rel_ than for L5B-POm in rats (Table 1; Groh et al., 2008; Seol and Kuner, 2015). We thus conclude that the PIR-MD synapses investigated here are of the “class I” type.

**Table 1:** **Comparison with other giant synapses**.

Synapse	Species	20–80% rise time [ms]	Decay constant [ms]	Amplitude [pA]	RRP [pA]	P_rel_ [%]	Recovery constant [ms]	Steady-state STD at 50 Hz [%]
PIR-MD	Mouse	0.54 ± 0.08	2.25 ± 0.97	391 ± 522	862 ± 947	50 ± 22	1340 ± 290	18
L5B-POm^a^	Mouse	0.80 ± 0.05	3.50 ± 0.2	257 ± 33	448 ± 46	51 ± 7	1: 15 ± 4	20^+^
							2: 195 ± 187	
L5B-POm^b^	Rat	0.43 ± 0.17	1.23 ± 0.27	3330 ± 1450	4100 ± 1700	80 ± 9	1: 27	14 ± 7
							2: 423	

*RRP, readily releasable pool; P_rel_, release probability; ^a^ = Seol and Kuner (2015), ^b^ = Groh et al. (2008), ^+^ = inferred from figure. Mouse recordings done at room temperature, rat recordings at 35°C*.

### Postsynaptic Activity

Only in a single out of eight recordings analyzed was the postsynaptic current sufficiently strong to reliably evoke APs. Postsynaptic APs were also not observed in any of the recordings that were excluded from analysis. Thus, the low probability of suprathreshold responses found here (approximately 12%) is likely an overestimate and lower than that found in other reports. Seol and Kuner (2015) report that 21% of all EPSCs were large enough to trigger postsynaptic spikes. In case of the retinogeniculate synapse 46 ± 16% of retinal APs are followed by a thalamic AP in rhesus monkey (Sincich et al., 2007). Budisantoso et al. (2012) found that large EPSCs always trigger postsynaptic APs, while small (<500 pA) EPSCs fail more often as the membrane potential decreases. In other studies the probability is not explicitly stated or apparent from the data, but it is obvious that also not all EPSCs trigger APs (Turner and Salt, 1998; Li et al., 2003; Reichova and Sherman, 2004).

The postsynaptic AP generation is a response property of the postsynaptic neuron. Mainly the I_T_ currents shape the responses at low membrane potentials. A Ca_v_3.1 knock-down study in relay cells of the POm nucleus clearly showed that the postsynaptic spiking at hyperpolarized membrane potentials is eliminated, while at deperpolarized potentials the “tonic” relay function is fully expressed (Seol and Kuner, 2015). The same study also establishes that the EPSC amplitude and number of postsynaptic APs does not correlate. It is therefore the interplay of presynaptic transmitter release and postsynaptic receptor and channel profile that determines the input-output function in thalamic relay cells.

The apparent lack of postsynaptic AP generation is therefore not directly a property of the synapse itself, but may reflect a distinct property of the MD neurons.

### Function of the Connection Between PIR and MD

The results presented here clearly indicate that most “class I” PIR-MD synapses are by themselves not sufficient to evoke APs in the MD. This may suggest that multiple such inputs need to be integrated at the dendrite or soma. In this scenario, the MD neurons would function as an integrator rather than a mere relay for information from the PIR to the prefrontal cortex. Whether this is causally linked to the fact the circuit for olfactory information processing is different to other sensory modalities, in the sense that the thalamus is downstream of the primary olfactory cortex, while usually the thalamus operates before the primary sensory cortices, remains to be determined. However, guided by the idea that class I synapses represent the principal information we conclude that at least a subset of mediodorsal thalamic neurons are important for olfactory information processing.

## Author Contributions

PP: design of project, animal injections, electrophysiology, analysis and 3D reconstruction of EM images, interpretation, writing the manuscript. HH: preparation, acquisition and analysis of EM images, revising draft manuscript. TK: design of project, supervision of research, writing the manuscript.

## Funding

Thomas Kuner acknowledges funding from the Deutsche Forschungsgemeinschaft (DFG) Priority Program 1392 and DFG Collaborative Research Center 636. The publication of this article was supported by the DFG and Heidelberg University within the “Open Access Publishing” funding program.

## Conflict of Interest Statement

The authors declare that the research was conducted in the absence of any commercial or financial relationships that could be construed as a potential conflict of interest.
