# Supplementary material for: Ultrastructural and Functional Properties of a Giant Synapse Driving the Piriform Cortex to Mediodorsal Thalamus Projection
Source: Front Synaptic Neurosci. 2017 Jan 31;9:3. doi: 10.3389/fnsyn.2017.00003 (PMC5281591; doi:10.3389/fnsyn.2017.00003)
Supplement: Supplementary file 1 [file DataSheet_1.docx]

Supplementary Material

Ultrastructural and Functional Properties of a Giant Synapse Driving the Piriform Cortex to Mediodorsal Thalamus Projection

Patric Pelzer, Heinz Horstmann, Thomas Kuner*

*** Correspondence:** Thomas Kuner: kuner@uni-heidelberg.de

**
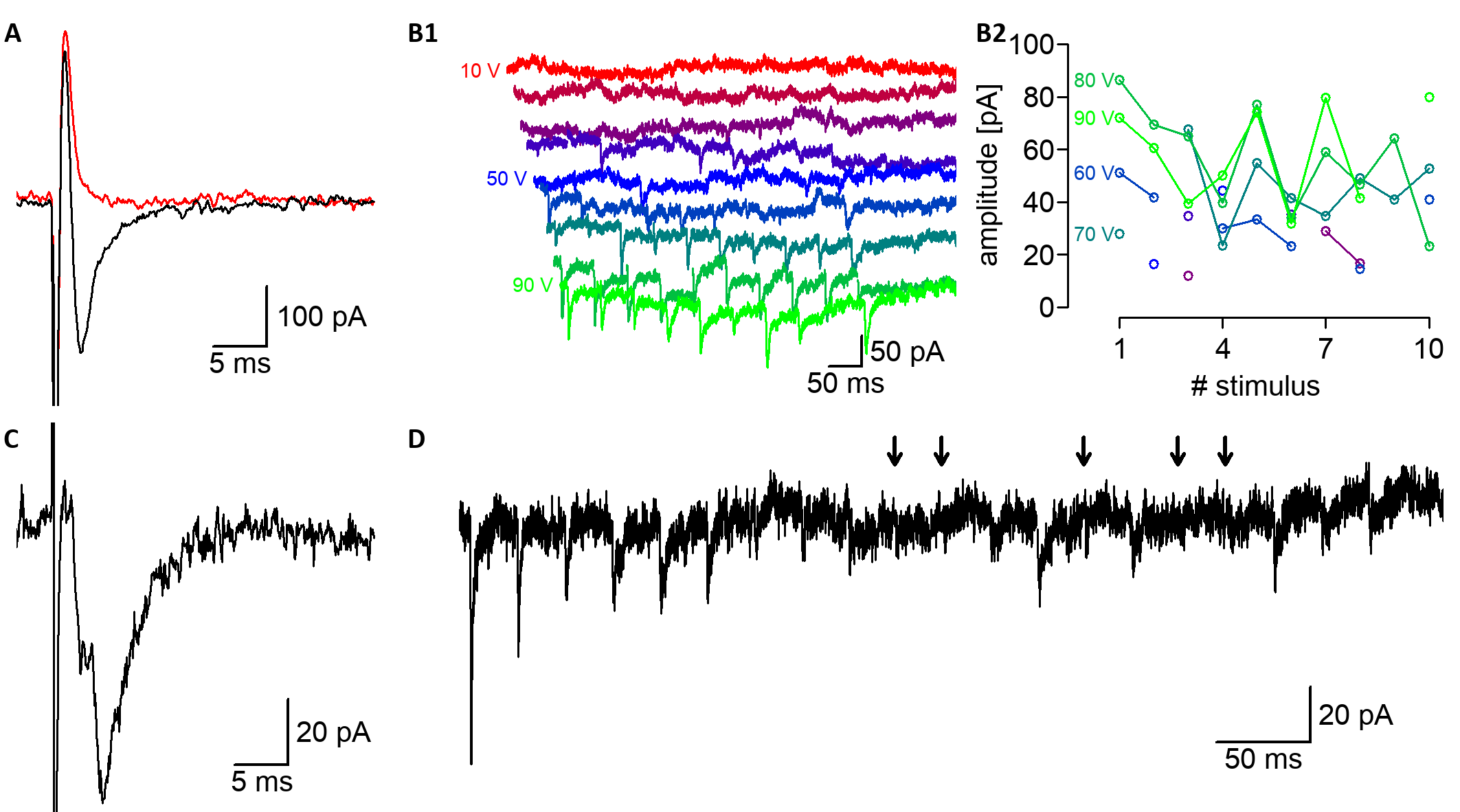
**

**Supplementary Figure 1 |** **Examples for exclusion criteria.** **(A)** The rise phase of the EPSC is not clearly separated from the stimulus artifact. (red: lower stimulus intensity - no EPSC, black: higher intensity - with EPSC) (**B1**) Example traces of postsynaptic responses to 10 stimuli at 20 Hz with increasing stimulation intensity (10 – 90 V). Stimulus artifacts were removed. (**B2**) Quantification of EPSC amplitudes for the 10 stimuli (color code as in B1). The amplitude is quite variable, but seems to scale with stimulus intensity. (**C**) Example trace violating the “monosynapse” criterion: a clear second component adds with a short delay. (**D**) An example trace with multiple failures (indicated by the arrows). Stimulus artifacts were removed.


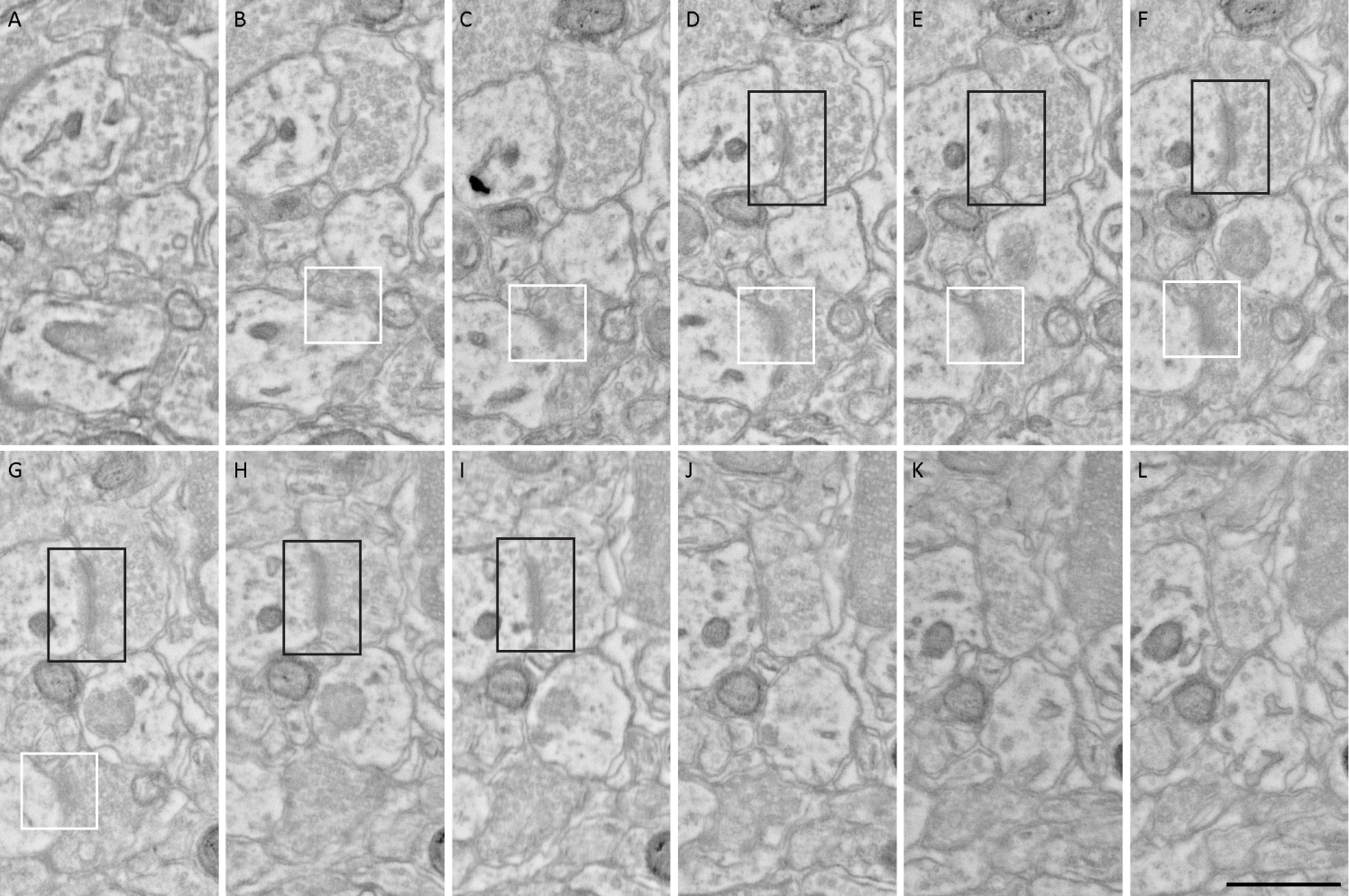


**Supplementary Figure 2 | Active zones. (A-L)** Consecutive electron microscopic sections of unlabeled MD tissue. The black box frames a clearly identifiable active zone with the postsynaptic density present. The white box frames a less clearly prominent active zone. The active zone is most likely not cut perpendicular to the synaptic cleft and therefore smeared out and has an apparent lower contrast. Scale bar = 500 nm


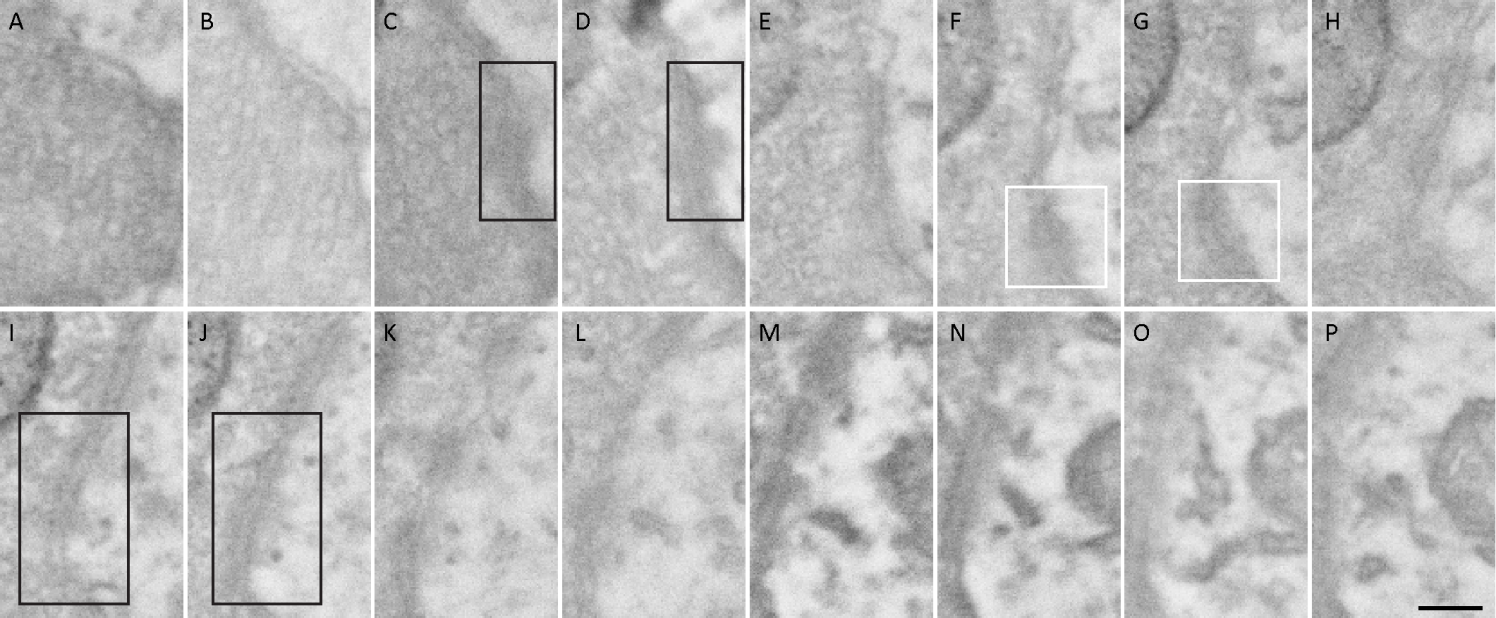


**Supplementary Figure 3 | Potential active zones in a labeled RL-type synapse. (A-P)** Consecutive electron microscopic sections of an identified PIR-MD RL-type bouton. Potential postsynaptic densities are apparent in only a few sections and in not more than 2 consecutive sections (two sets of black frames and a white frame). The active zone cannot be unequivocally identified as such, as the potential gray intensities match the electron-dense precipitate used to unequivocally confirm the PIR identity. Scale bar = 100 nm.


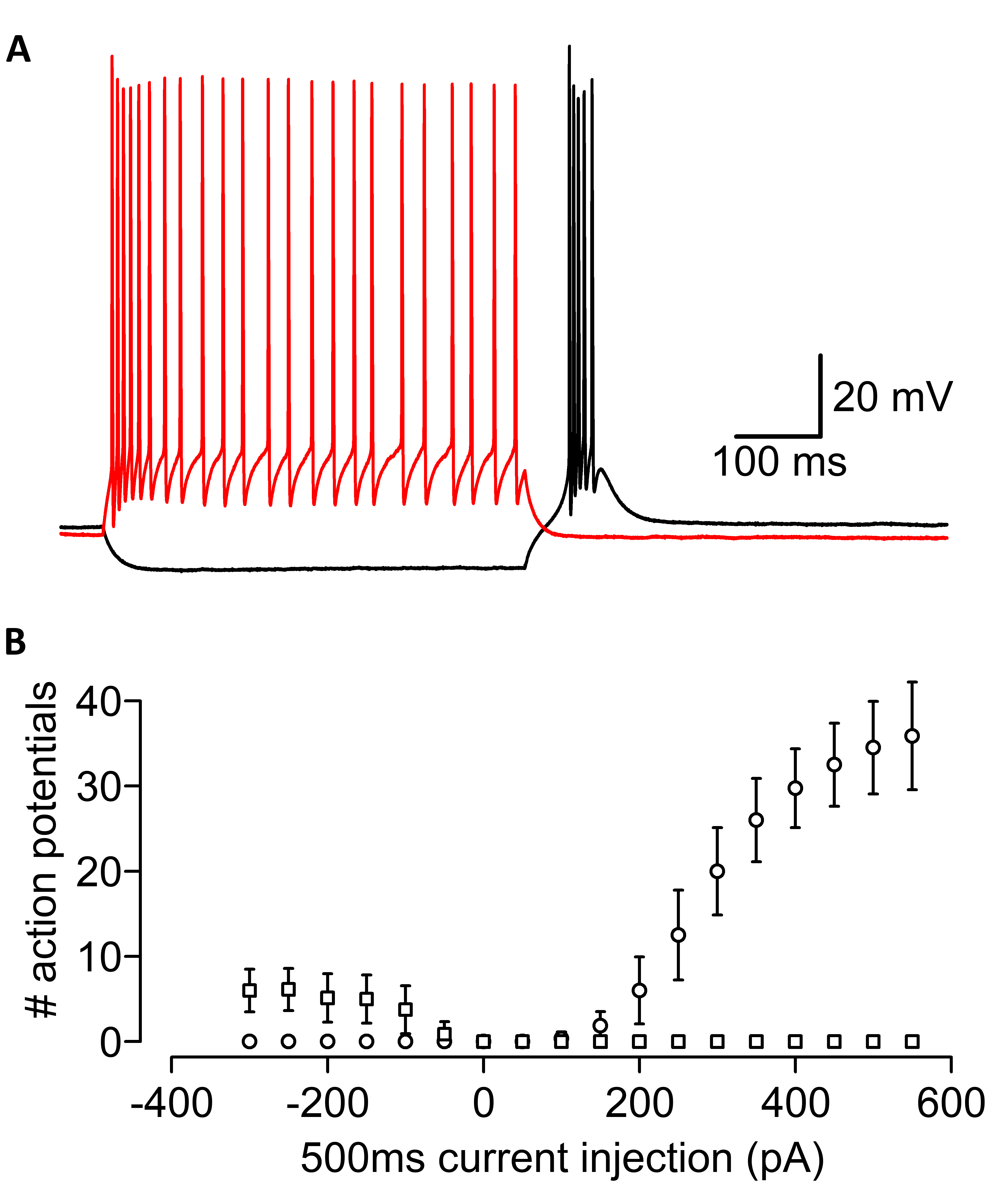


**Supplementary Figure 4 | Tonic and burst mode in mediodorsal thalamic relay neurons.** **(A)** Typical responses to -150 and +300 pA current injection. **(B)** Quantification of the number of action potentials (APs) during (○) and after (□) the current injection (n = 8 neurons).


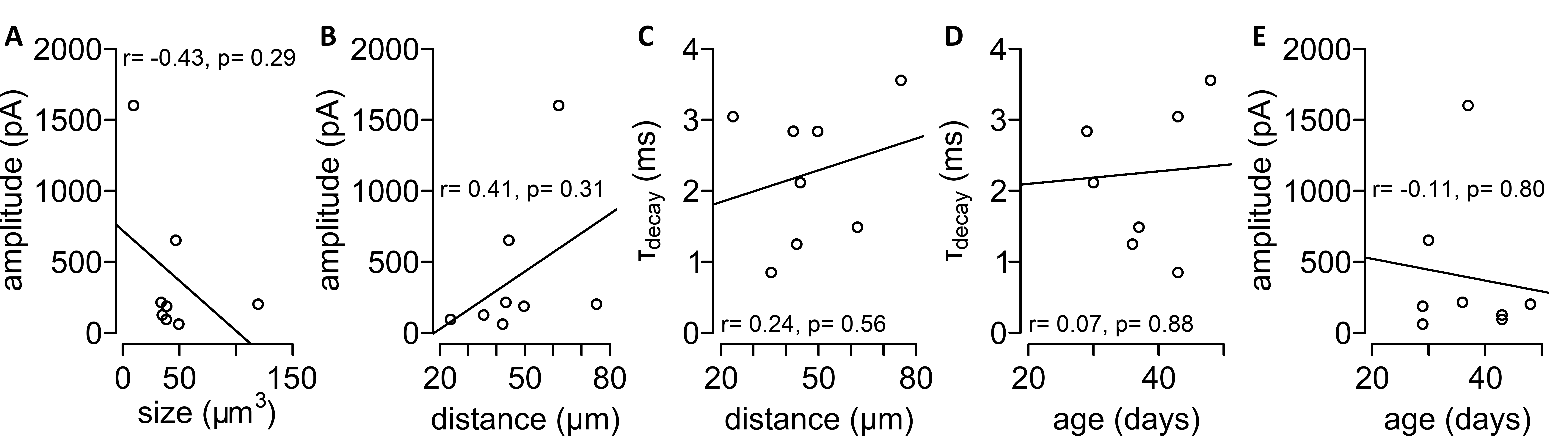


**Supplementary Figure 5 | Extended Characteristics of Evoked Postsynaptic Currents of the PIR-MD Synapses.** **(A)** The EPSC amplitude does not correlate with the size of the synapse. **(B)** The distance between the synapse and the soma does not affect the amplitude or **(C)** the decay kinetics. **(D-E)** Neither do the amplitude and decay time change with the age of the animal.

**
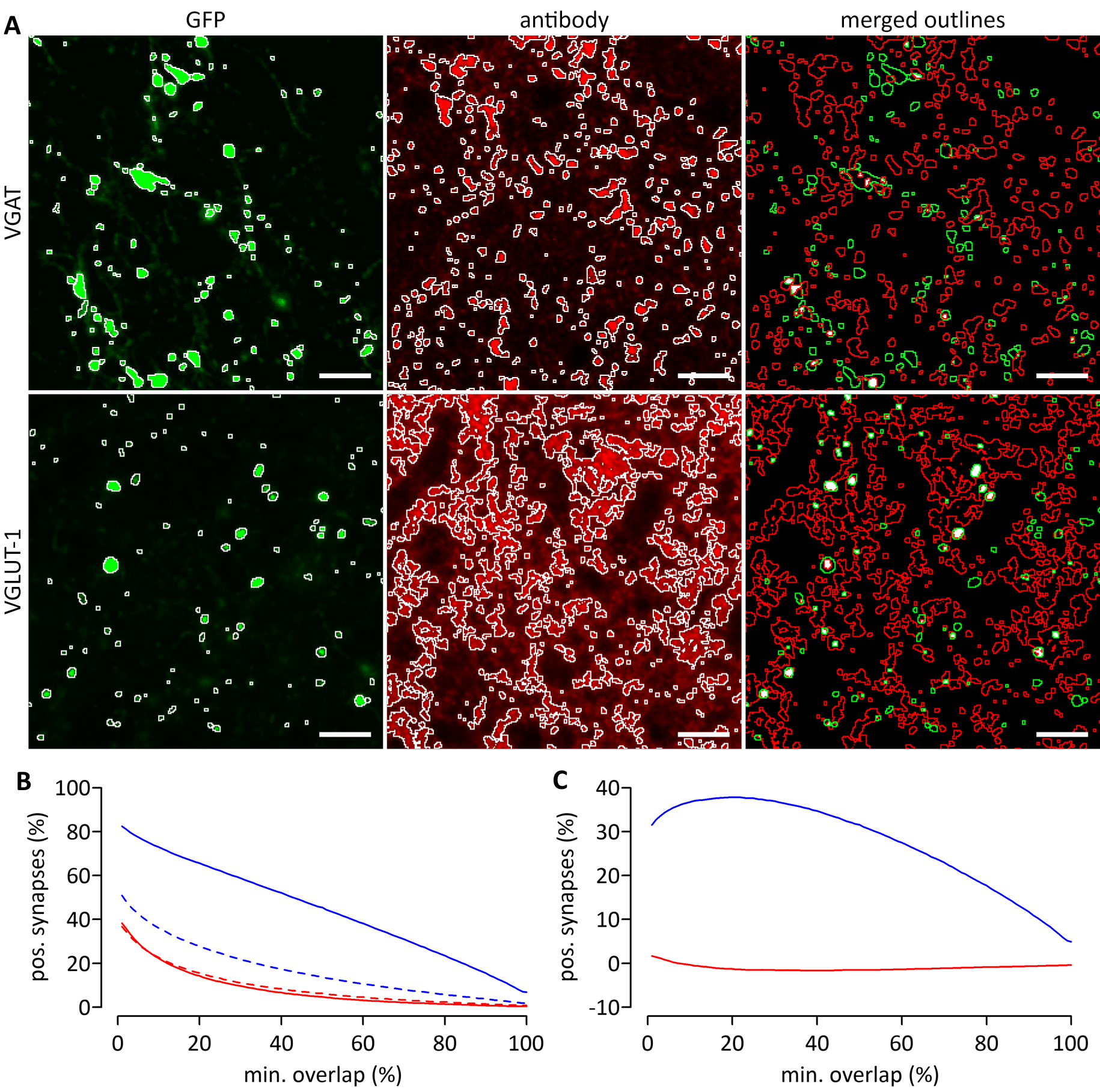
**
 **Supplementary Figure 6 | Superposition of GFP-labeled PIR synapses in the MD nucleus with antibody stainings against VGAT and VGLUT-1.** **(A)** Single confocal image planes taken from a stack. Upper row: staining against VGAT; lower row: staining against VGLUT-1. Left column: GFP expression demarcating terminals originating from PIR. Images were thresholded to obtain binary representations (outlined by white lines); middle column: antibody signal, binary signal outlined in white; right column: merged color-matched outlines obtained from the panels to the left with overlap filled in white. **(B)** Percentage of synapses positive for the antibody signal for different extents of overlap between synapse volume and antibody signal (solid blue line = VGLUT-1, solid red line = VGAT). The percentage for the controls (averaged concurrence when images are rotated by 90°, 180° and 270° against each other) is shown by the dashed lines. **(C)** Residual positive synapses after subtraction of the control. Hence, VGLUT-1 shows a clear colocalization with GFP-labeled PIR synapses while VGAT does not. Images show representative examples of 13 (VGLUT-1) and 10 (VGAT) image stacks taken from 2 (VGLUT-1) and 3 (VGAT) mice. Scale bars 10 µm.

**Methods to Supplementary Figure 6**

Mice were deeply anesthetized with isoflurane and transcardially perfused with 20 ml PBS followed by 20 ml 4 % PFA. Post-fixated (4 % PFA) brains were sliced at a thickness of 50 µm on a vibratome. Slices were permeabilized and unspecific binding sites were blocked for 30 ‑ 45 min in blocking buffer (VGLUT-1: 5 % NGS, 1 % BSA, 0.5 % Triton X‑100 in PBS; VGAT: 5 % NGS, 1 % BSA, 0.3 % Triton X‑100, 0.1 % cold fish skin gelantin in PBS). Then they were washed in vehicle solution (VGLUT-1: 2.5 % NGS, 0.2 % BSA, 0.5 % Triton X‑100 in PBS; VGAT: 5 % NGS, 1 % BSA, 0.3 % Triton X‑100, in PBS) for 10 min. The primary antibody (VGLUT-1 (AB5905, Millipore, 1:1000) and VGAT (131004, Synaptic Systems, 1:250), diluted in the vehicle solution, was applied overnight at 4 °C. The next day, the tissue was washed 3 times in vehicle for 10 min each. The secondary antibody (Alexa-594-coupled, A-11076, Invitrogen, 1:1000), also diluted in vehicle solution, was applied for 90 ‑ 120 min under light protection. The tissue was finally washed twice in vehicle solution and twice in PBS for 10 min each. Confocal imaging was done on a Leica TCS SP5 using a 63x APO objective. Finally, the confocal image stacks were binarized into background and signal of both channels, i.e. GFP signal and antibody staining (**Figure S6A**). From the binary image stacks we extracted for each synapse its overlap, i.e. the percentage of the volume that is also positive for the antibody. In order to quantify the percentage of synapses positive for either marker, individual synapses were scored as positive, if its overlap with the antibody reached the threshold in the range from 1 to 100 % (**Figure S6B**). A 90°, 180°, and 270° rotation of one channel served as a control for overlap by chance. Finally the data was corrected for the control values (**Figure S6C**).
